# Fumarate hydratase inhibition activates innate immunity via mitochondrial nucleic acid release

**DOI:** 10.1002/mco2.314

**Published:** 2023-07-06

**Authors:** Xiang Li, Fangfang Zhou, Linghui Zeng

**Affiliations:** ^1^ Key Laboratory of Novel Targets and Drug Study for Neural Repair of Zhejiang Province School of Medicine Hangzhou City University Hangzhou China; ^2^ MOE Laboratory of Biosystems Homeostasis and Protection and Innovation Center for Cell Signaling Network Life Sciences Institute Zhejiang University Hangzhou China; ^3^ Institutes of Biology and Medical Science Soochow University Suzhou China

1

Recently, two back‐to‐back studies (Zecchini et al.^1^ and Hooftman et al.^2^) were published in *Nature*, illustrating the vital protective role of fumarate hydratase (FH) in innate immune responses. Their findings showed that FH inhibition induced the accumulation of fumarate, leading to the release of mitochondrial DNA (mtDNA) and mitochondrial RNA (mtRNA) respectively, which further increased the production of type‐I interferon (IFN) through activation of the DNA sensor cyclic guanosine monophosphate–adenosine monophosphate adenosine synthetase (cGAS) and RNA sensors toll–like receptor 7 (TLR7), retinoic acid–inducible gene I (RIG‐I), and melanoma differentiation–associated gene 5 (MDA5). Their work revealed the relationship between FH inhibition and mitochondrial nucleic acid release as well as innate immune activation, which was a crucial breakthrough in the crosstalk between metabolism and immunity.

FH is a key metabolic enzyme in the tricarboxylic acid (TCA) cycle and catalyzes the reversible hydration of fumarate to malate. FH deficiency in the cells leads to fumarate accumulation. High levels of fumarate, as a substrate or product, alter the balance between multiple enzymatic reactions, ultimately causing changes in cellular metabolism. Thus, the loss of FH results in profound metabolic changes. FH mutations have been implicated in the pathogenesis of various diseases, most typically hereditary leiomyomatosis and renal cell carcinoma (HLRCC), a cancer syndrome characterized by cutaneous uterine leiomyomas and papillary type II renal cell carcinomas (RCC), one of the most aggressive forms of renal carcinoma. FH loss and fumarate accumulation have been shown to inactivate core factors responsible for mitochondrial DNA replication and proofreading in HLRCC tumors. The release of mtDNA into the cytoplasm and extracellular environment activates a large number of different pattern recognition receptors and innate immune responses, including cGAS–stimulator of interferon genes (STING), toll–like receptor 9 (TLR9), and inflammasome formation, as well as powerful type‐I IFN responses. However, the contribution of FH to innate immunity via fumarate regulation remains unclear.

Zecchini et al.^1^ studied the acute response to loss of *Fh1* (the mouse orthologue of human *FH*) using a tamoxifen‐inducible *Fh1*‐deficient mouse model. They analyzed the mouse kidney transcriptome and found that inflammation and innate immune pathways were upregulated in *Fh1*‐deficient mice, which was less relevant to immune cells. Using transmission electron microscopy (TEM) and droplet digital PCR, they observed a time‐dependent increase in mtDNA in the cytosol combined with a progressive remodeling of mitochondrial morphology, which were caused by the loss of FH activity, but not the overall impairment of mitochondrial metabolism. Treatment with monomethyl fumarate, a cell‐permeable derivative of fumarate that increases fumarate levels, phenocopied the effects of FH deficiency, suggesting that fumarate is the main driver of innate immune response activation. Subsequently, they identified the cGAS–STING cascade as being responsible for the inflammatory response induced by fumarate. Furthermore, TEM imaging showed that mtDNA is released into the cytosol by mitochondria‐derived vesicles (MDVs), in which the endocytic accessory protein sorting nexin 9 (SNX9) plays a crucial role (Figure 1). Hooftman et al.^2^ investigated the effect of FH inhibition on mtRNA release in lipopolysaccharide (LPS)‐stimulated macrophages. They used an unbiased metabolomics approach to evaluate metabolic alterations induced by LPS stimulation and found that fumarate was significantly upregulated following LPS stimulation, mainly due to the upregulation of argininosuccinate synthase and increased aspartate‐argininosuccinate shunt. The FH levels remained unchanged in the early stages of LPS stimulation and were downregulated in later stages. Using pharmacological inhibitor fumarate hydratase–IN–1 (FHIN1) and *Fh1*‐deficient mice to inhibit FH induced substantial redox stress responses and significantly upregulated tumor necrosis factor signaling. Further, FHIN1 increased the production of interferon–β (IFN‐β) in the microphage stimulated by LPS, which may be dependent on mtDNA. Surprisingly, Hooftman et al.^2^ proved that the cGAS–STING or TLR9 DNA‐sensing pathways were not involved in this process. They subsequently confirmed that the FHIN1‐driven IFN response required mtRNA and its sensors, TLR7, RIG‐I, and MDA5. Disturbances in mitochondrial membrane potential (MMP) were also connected to the mtRNA release and IFN‐β induction. Mitochondrial membrane hyperpolarization and mtRNA release are endogenously activated during the late phase of LPS activation (Figure 1).

**FIGURE 1:**
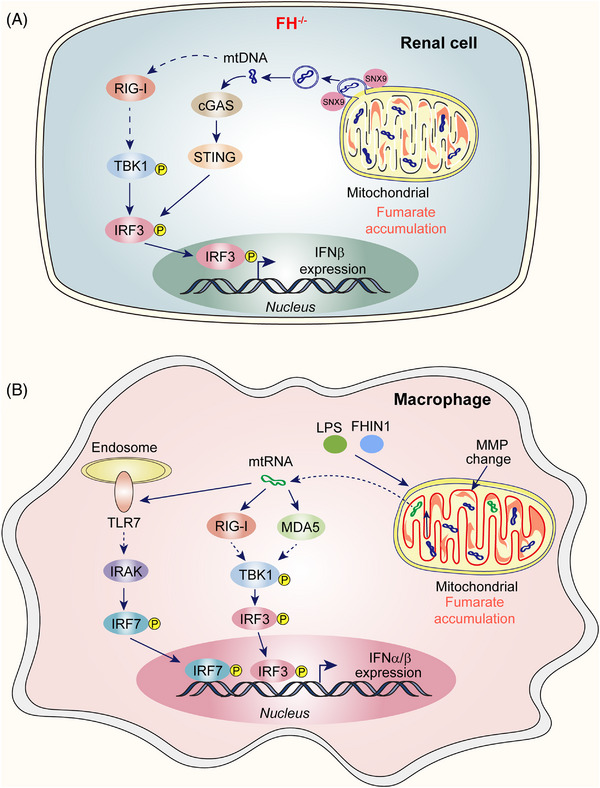
Role of fumarate hydratase (FH) in innate immune response. (A) Loss of FH in renal cells leads to the accumulation of fumarate in mitochondria and the disruption of mitochondrial morphology, which leads to the release of mitochondrial DNA (mtDNA) into the cytoplasm, where mtDNA activates the cGAS–STING pathway as well as RIG‐I and induces IFN‐β production. (B) LPS stimulation and FHIN1 treatment result in the suppression of FH, which leads to mitochondrial membrane hyperpolarization and mitochondrial RNA (mtRNA) release into the cytoplasm, where mtRNA activates TLR7, RIG‐I, and MDA5, followed by IFN‐α/β production.

Released mtDNA and mtRNA have been reported to activate cytosolic nucleic acid sensors to induce type‐I interferon production and inflammatory response.^3^, ^4^ Zecchini et al.^1^ and Hooftman et al.^2^ illustrated that FH deficiency induced an IFN response via the release of mtDNA or mtRNA in different physiological models, which is a novel crosstalk between mitochondrial metabolism and innate immunity. The accumulation of fumarate, a key metabolite of the TCA cycle, is likely to play a crucial role. This process has been thoroughly studied from different perspectives in the two articles, respectively. Zecchini et al.^1^ proposed that FH inhibition induces the SNX9‐dependent formation of mtDNA‐containing MDV, a novel mechanism by which fumarate drives mtDNA release. mtDNA was previously reported to be transmitted between tumor cells via extracellular vesicles,^5^ but the transport of mtDNA in MDVs was identified for the first time. Hooftman et al.^2^ found that FH inhibition resulted in the release of mtRNA by increasing the MMP. There are few reports on the mechanism of mtRNA release, mainly the observation of mtRNA release due to the B–cell lymphoma–2 associated X, apoptosis regulator/B–cell lymphoma–2 antagonist/killer 1‐mediated permeabilization of the outer membrane during programmed cell death.^4^ Therefore, the discovery of Hooftman et al. is a breakthrough for the study of the release mechanism of mtRNA. The analysis of the crosstalk between mitochondrial metabolism and innate immunity is also a highlight of both researches. Zecchini et al.^2^ comprehensively analyzed the inflammatory response triggered by FH inhibition, while Hooftman et al.^2^ evaluated the changes of aspartate‐argininosuccinate shunt by LPS stimulation and the metabolic rewiring caused by FH inhibition. FH inhibition using pharmacological inhibitor FHIN1 induced alteration of TCA cycle metabolites, modulation of cytosolic processes, and substantial redox stress responses.

Notably, the two studies did not agree on how mtDNA and mtRNA induce IFN production after release, possibly because of the differences in the models used. Zecchini et al.^1^ verified that mtDNA release resulting from FH inhibition activates the IFN response via the cGAS–STING pathway in kidney cells, whereas Hooftman et al.^2^ observed that the cGAS–STING pathway was redundant for the IFN response triggered by FH inhibition in LPS‐stimulated macrophages. The reasons for these differences require further investigation. Moreover, there are still many unknowns about the release mechanism of mitochondrial nucleic acids. It is a fascinating question whether mtRNAs exist within the MDVs proposed by Zecchini et al.^1^ and whether changes in MMP affect mtDNA release. Combining models from both groups may lead to a more comprehensive understanding of this process. Besides, Zecchini et al.^1^ did not explain how mtDNA is released from MDVs, which is crucial for the recognition of cGAS.

In brief, these two studies revealed that FH inhibition activates innate immune or inflammatory responses via the release of mtDNA or mtRNA. Studies on renal cell carcinoma and LPS‐stimulated macrophages have demonstrated the extensive physiological and pathological background and application potential of fumarate accumulation driven by FH inhibition. Overall, both studies elucidate the connection between mitochondrial metabolism and the innate immune response and provide guidance on clinical strategies for related diseases.

## AUTHOR CONTRIBUTIONS

X. L. conceived and drafted the manuscript, and drew the figure. F. Z. and L. Z. discussed and revised the manuscript. All authors have read and approved the article.

## CONFLICT OF INTEREST STATEMENT

The authors declare no conflict of interest.

## ETHICS STATEMENT

Not applicable.

## Data Availability

Not applicable.
